# Coastal and nearshore sediment data along the eastern coastal zone of Bangladesh of the northern Bay of Bengal

**DOI:** 10.1016/j.dib.2023.109028

**Published:** 2023-03-01

**Authors:** Md. Zakaria, Mst Tania Islam, Sayeed Mahmood Belal Haider

**Affiliations:** a Geological Oceanography Division, Bangladesh Oceanographic Research Institute, Upazila (Sub-district): Ramu, District: Cox's Bazar, Post Code: 4730, Bangladesh; b Oceanographic Data Center, Bangladesh Oceanographic Research Institute, Upazila (Sub-district): Ramu, District: Cox's Bazar, Post Code: 4730, Bangladesh; c Bangladesh Oceanographic Research Institute, Upazila (Sub-district): Ramu, District: Cox's Bazar, Post Code: 4730, Bangladesh

**Keywords:** Sediment texture, Grain size distribution, Cox's Bazar, Teknaf, Saint Martins's Island, Sedimentary characteristics

## Abstract

A seafloor sediment dataset of 42 samples has been collected from the coastal and nearshore areas of the northern Bay of Bengal, which is situated in the southern part of the eastern coastal zone of Bangladesh. From February 2018 to March 2019, Van Veen grab samplers were employed to gather samples at a depth of 4 m and 23 m. The unconsolidated bottom surface has been sampled, taking bathymetry and fishing boat accessibility into account. Using the dry sieving technique, grain size distribution analysis was performed on the obtained sediments. The retrieved dataset was measured to identify seafloor sediment grain size distribution and determine the sediment dynamics. This data can be used to delineate sediment characteristics, depositional processes, and the environment.

**Specifications Table**

**Table:** 

Subject	Earth-Surface Processes

Specific subject area	Sedimentology, Marine Geology, Coastal Geomorphology
Type of data	Table Image Graph Figure Map
How the data were acquired	A total of 42 sediment samples were collected from the seafloor. The samples were collected using a Van Veen grab sampler. Sediment sampling was conducted with the goal of delineating the coastal and nearshore processes, considering depth and distance from the shoreline. The survey was conducted using local fishing trawlers/boats, so the depth and distance were based on safety considerations and trying to maintain a grid pattern of data collection using spot location with GPS. Collected sediment samples were taken to the laboratory, where different measurements of sieve shaking were conducted. ASTM sieve mesh sizes 18, 35, 60, 120, and 230 were used to separate the sediment into various fractions such as very coarse, coarse, medium, fine, and very fine sand, as well as mud (which passed through the 230 mesh sizes). The separated samples were then measured using a laboratory analytical balance. The statistical parameters, including histograms and cumulative curves, were calculated using MS Excel software. The cumulative curve was used to extract the phi size values of different percentages, which were then used to calculate the statistical parameters of grain size, including mean size, sorting, skewness, and kurtosis, using MS Excel software.
Data format	Raw Analyzed
Description of data collection	Seafloor sediment was collected between February 2018 and March 2019. The shallowest sample has been retrieved from a depth of 4 m and the deepest sample from a depth of 23 m. Grain size analysis was conducted in the laboratory.
Data source location	Eastern Coast of Northern Bay of Bengal, Bangladesh. From the southernmost border of Bangladesh (Saint Martin's Island) to the northward Maheshkhali Channel of the Cox's Bazar District- within the coastal and nearshore area (Territorial sea)
Data accessibility	**Mendeley Data** Coastal and nearshore sediment samples of the eastern coastal zone of Bangladesh https://data.mendeley.com/datasets/k3p7sn6zw3 **doi:**10.17632/k3p7sn6zw3.3

## Value of the Data

• Surficial unconsolidated sediment of the seafloor of the northern Bay of Bengal, Bangladesh was poorly or never studied prior to the collection and analysis of this shallow marine dataset.
• The coastal and nearshore sediment data can be used for sediment dynamics, depositional processes, depositional environment, and engineering studies for the benefit of policymakers, researchers, consultants, and stakeholders to make their decisions.
• There is the longest uninterrupted sandy sea beach, named Cox's Bazar sea beach, situated along the sampling zone. Nowadays, the beach area is undergoing erosion in the popular tourist spot. The dataset can be used for the reconstruction and nourishment of the beach.
• The dataset can help with the potential delineation of sediment transportation, distribution, erosion, and deposition in the area.

## 1 Objective

The dataset was created to study grain size characteristics, sediment depositional processes, sediment transportation, and the identification of the depositional environment of Bangladesh's eastern coastal zone, which is part of the northern Bay of Bengal. This data article will be used to determine the proper results by utilizing the insights output of the data for the research article. Also, the dataset will be an important tool for understanding the physical, chemical, and biological processes that shape coastal and nearshore ecosystems and for developing effective strategies for coastal management and conservation. This dataset can be used to study the impact of sedimentation on water quality in coastal areas, design and implementation of coastal habitat restoration, understand the processes of coastal erosion and sediment transport, identification of suitable sediment sources of beach nourishment and, source and level of pollution monitoring (such as distribution of heavy metals, pesticides, and microplastics). Besides this dataset can also be used for seafloor mapping and exploration, geohazards assessment, and, engineering applications.

## 2 Data Description

**Fig. 1:**
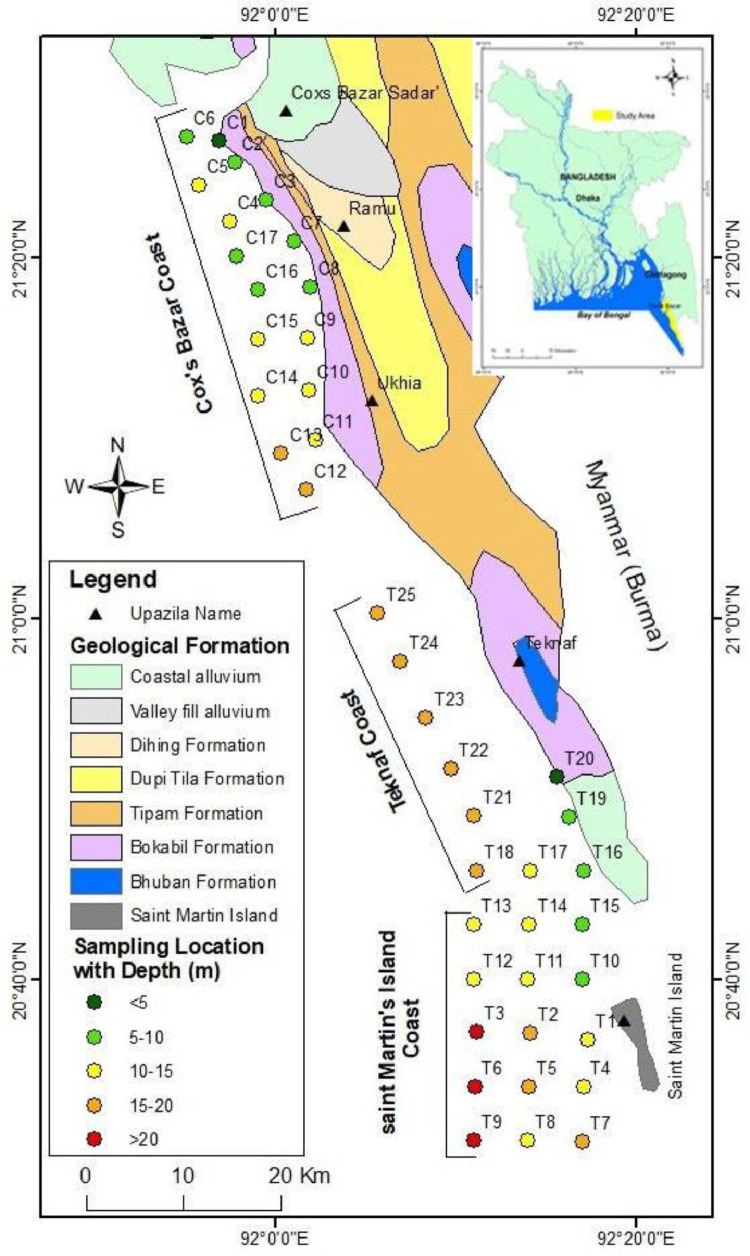
Simplified surface geological map of the eastern coastal zone of Bangladesh (modified from Alam et al. [1] and Hossain et al. [2]), and sampling location with bathymetry (depth in meters) at the coastal and nearshore area of the northern Bay of Bengal (Cox's Bazar coast, Teknaf coast, and Saint Martin's Island coast).

A total of 42 samples have been collected from the eastern coastal zone of Bangladesh, where 17 samples are from the Cox's Bazar coast, 10 samples from the Teknaf coast, and 15 samples from the Saint Martin's Island coast (Fig. 1). The survey covered approximately 1300 sq. km of the coastal and nearshore area of the eastern coastal zone of Bangladesh.

On the Cox's Bazar coast, 17 samples were collected, of which 1 (C1) was found at 4 m depth, 7 (C2, C3, C6, C7, C8, C16, and C17) between 5 and 10 m, 7 (C4, C5, C9, C10, C14, and C15) between 10 and 15 m, and 2 (C12 and C13) between 15 and 20 m depth. On the Teknaf coast, 10 samples were collected, of which 6 (T18, T21, T22, T23, T24, and 25) were retrieved from 15 to 20 m depth, 2 (T16, and T19) from 5 to 10 m, 1 (T17) from 13.50 m, and 1 (T20) from 5 m depth. Similarly, 15 samples were collected from Saint Martin's Island coast, of which 3 (T3, T6, and T9) are from 20 to 23 m in depth, 3 (T2, T5, and T7) are from 15 to 20 m, 7 (T1, T4, T8, T11, T12, T13, T14, and T17) are from 10 to 15 m, and 2 (T10, and T15)) are from 5 to 10 m in depth. The lowest depth sample retrieved from the Cox's Bazar coast was at 4 m depth (C1), and the highest depth sample retrieved from Saint Martin's Island coast was at 23 m depth (T9) (Fig. 1).

**Fig. 2:**
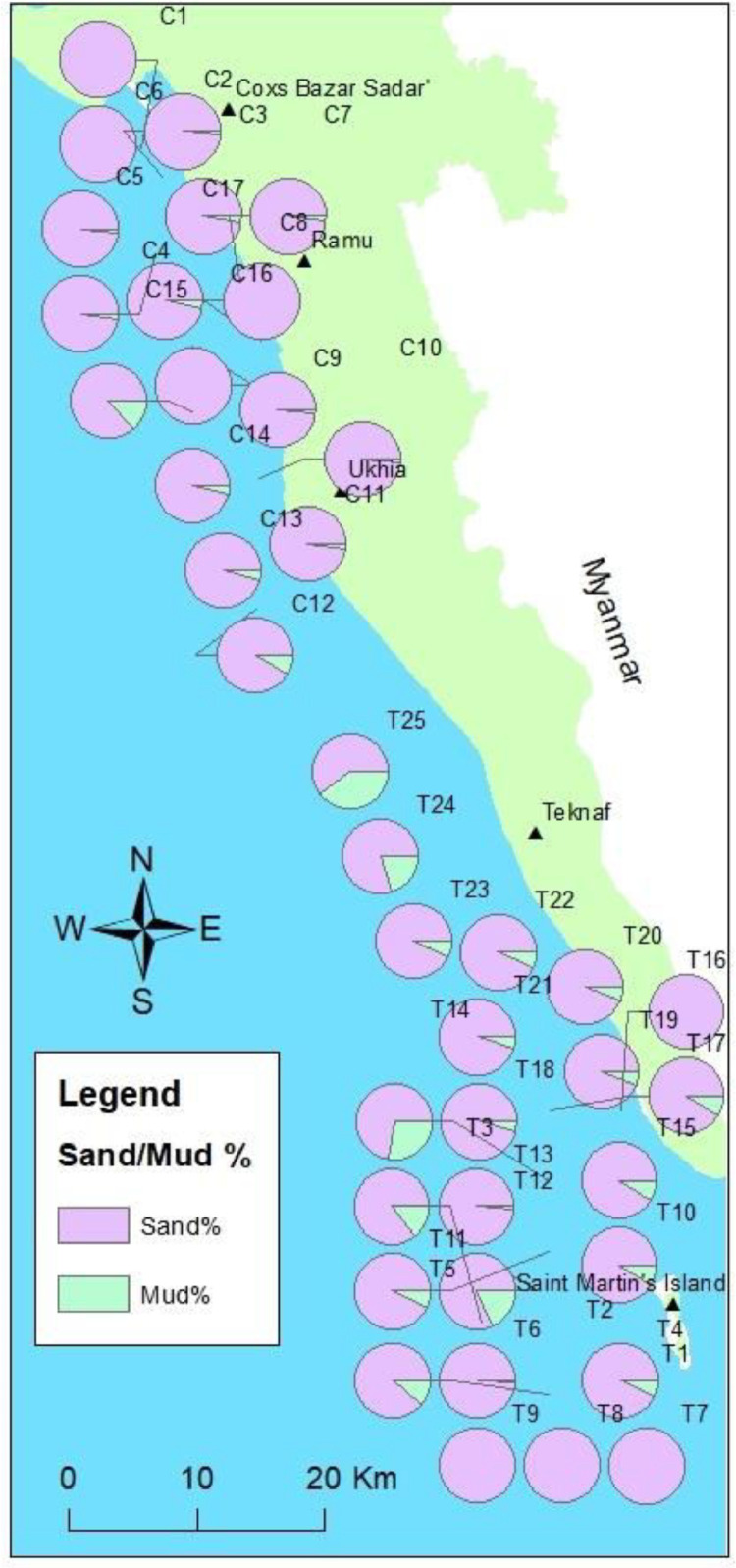
Percentage pie diagram map of sand and mud in each sampling location.

The sample collected from the nearshore area shows a high amount of sand (average 92.99%) and a low amount of mud (silt and clay) (average 7.01%). On the Cox's Bazar coast, all of the samples (except sample C15) have a higher percentage of sand than all of the samples (more than 98%). On the Cox's Bazar coast, only one sample (C15) contains 13.34% of mud and the rest of the samples show less than 5% of mud, except for C12 (Fig. 2b). The average sand is 97.06%, and mud is 2.94% on the Cox's Bazar coast. On the Teknaf coast, sample T16 shows more than 99% sand, and samples T24 (60%) and T25 (79%) show less sand. The range of mud of the sample T17–T23 is about 3%–8%, and sand is about 91%–96%. The mud content is higher in Samples T24 and T25, with a maximum of 39.67%. The average sand is 89.61% and mud is 10.93% along the Teknaf coast. Here, sand (2 mm to 0.063 mm in size) particles are typically larger in size than mud (<0.063 mm in size) particles, and sieve analysis used to distinguish between them based on their size differences [3].

The maximum sand found in the sample T7 (99.99%), where T6, T8, T9, and T13 show more than 98% of sand in the Saint Martin's Island coast samples. Samples T1, T2, T5, T12, and T14 show a lower percentage of sand (70–90%) and a higher amount of mud (10–30%). Sample T14 contains 72% sand and 27% mud. On Saint Martin's Island coast, the average sand is 90.61%, and the average mud is 9.36%. The average value shows that the Cox's Bazar coast contains a higher amount of sand and a lower amount of mud than the Teknaf coast, which shows a higher amount of mud and a lower amount of sand. But as per distribution, Saint Martin's Island coast shows a higher percentage of mud and a lower percentage of sand than the Teknaf coast (Fig. 2b).

**Fig. 3:**
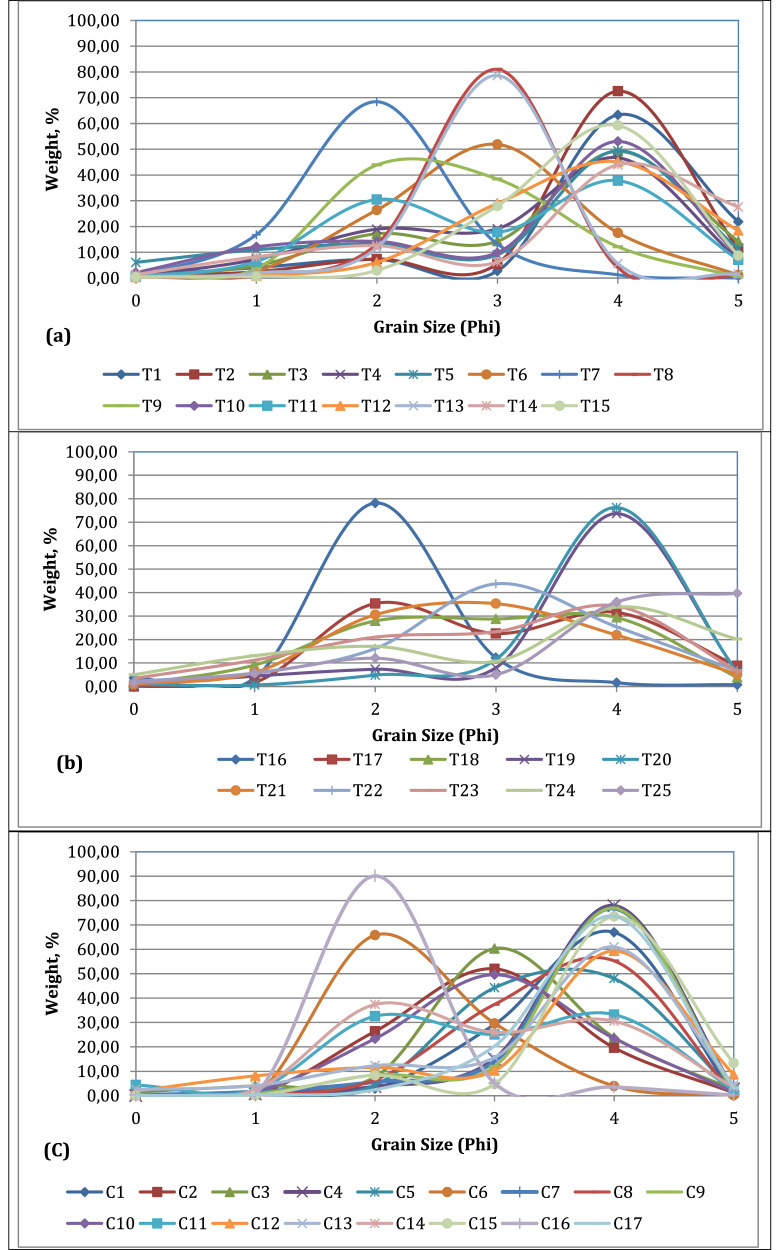
Frequency curve of the sediment of (a) Saint Martin's Island coast, (b) Teknaf coast, (c) Cox's Bazar coast samples.

The frequency curve is used to determine the modal nature through histogram construction [4]. On the Cox's Bazar coast, the frequency curve shows the dominant unimodal nature of the sediment. The sediment in samples C11 and C14 exhibits a wide range of bimodal characteristics. Samples C6 and C16 show a sharp peak of frequency at the 2 mm phi size in the graph, and samples C3, C2, and C10 show a standard peak at 3 mm phi size, where C1, C4, C9, C12, C13, and C17 show a sharp peak in the frequency curve at 4 mm of phi size (Fig. 3a).

The frequency curve of the Teknaf Zone shows the dominant bimodal nature of the sediment. Sample T16 shows unimodal with a 2 mm phi size, and samples T19 and T20 show unimodal with a 4 mm phi size of sediment. T22 shows a unimodal but flat peak in the frequency curve (Fig. 3b). Samples T3, T4, T5, T11, and T13 from Saint Martin's Island coast are bimodal, with the dominant peak at 4 mm phi size. T9 has a flat peak, T6 has a wide unimodal peak, and T7 has a unimodal with 2 mm of phi size sediment. The T8 and T13 show a sharp peak with a 3 mm phi size due to the unimodal nature of the sediment. T2 and T15 show unimodal sediment with a phi size of 4 mm (Fig. 3c).

**Fig. 4:**
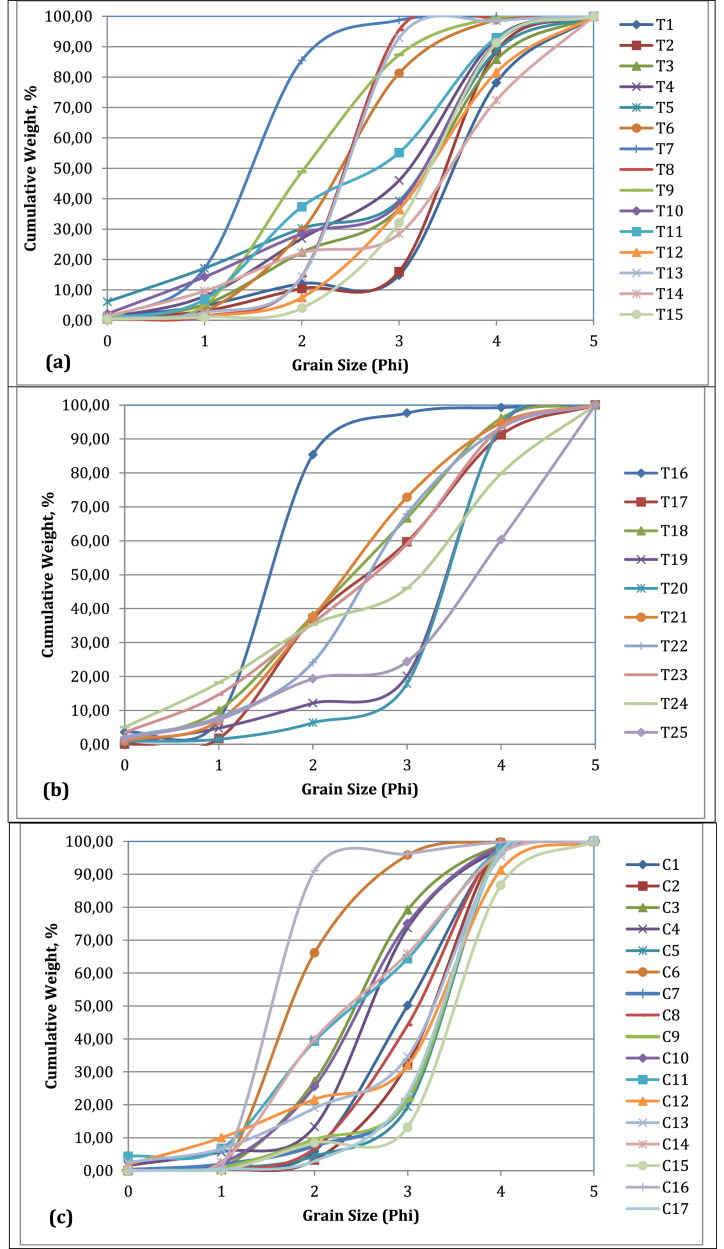
Cumulative curve of the sediment of (a) Saint Martin's Island coast, (b) Teknaf coast, (c) Cox's Bazar coast samples.

Grain size (phi scale) plotted against cumulative weight percentage generates a grain size cumulative curve, which is the most useful curve for grain size analysis [4]. Generally, the grain size cumulative curve generates an S-shaped curve. On the Cox's Bazar coast, the cumulative curve shows a steep slope. But C16 shows a very steep slope with medium-grain sand and C6 shows a steep slope with medium to fine-grain sand. Samples C4, C7, C9, C15, and C17 show a steep slope curve with a fine sand size distribution (Fig. 4c).

The majority of the sediment in the Teknaf Zone cumulative curve has a very gentle slope, with only sample T16 showing a very steep curve with medium sand size. Sample T13 shows a steep curve with fine sand size, whereas samples T19 and T20 show steep slopes with very fine sediment (Fig. 4b). The sediment of Saint Martin's Island coast shows a gentle slope in the cumulative curve where T7, T8, and T13 show steep slopes of medium to fine-grain sand. T9 shows a gentle slope with medium to fine-grained sand. Other samples of Saint Martin's Island coast show a gentle slope with fine to very fine sediment (Fig. 4a).

**Fig. 5:**
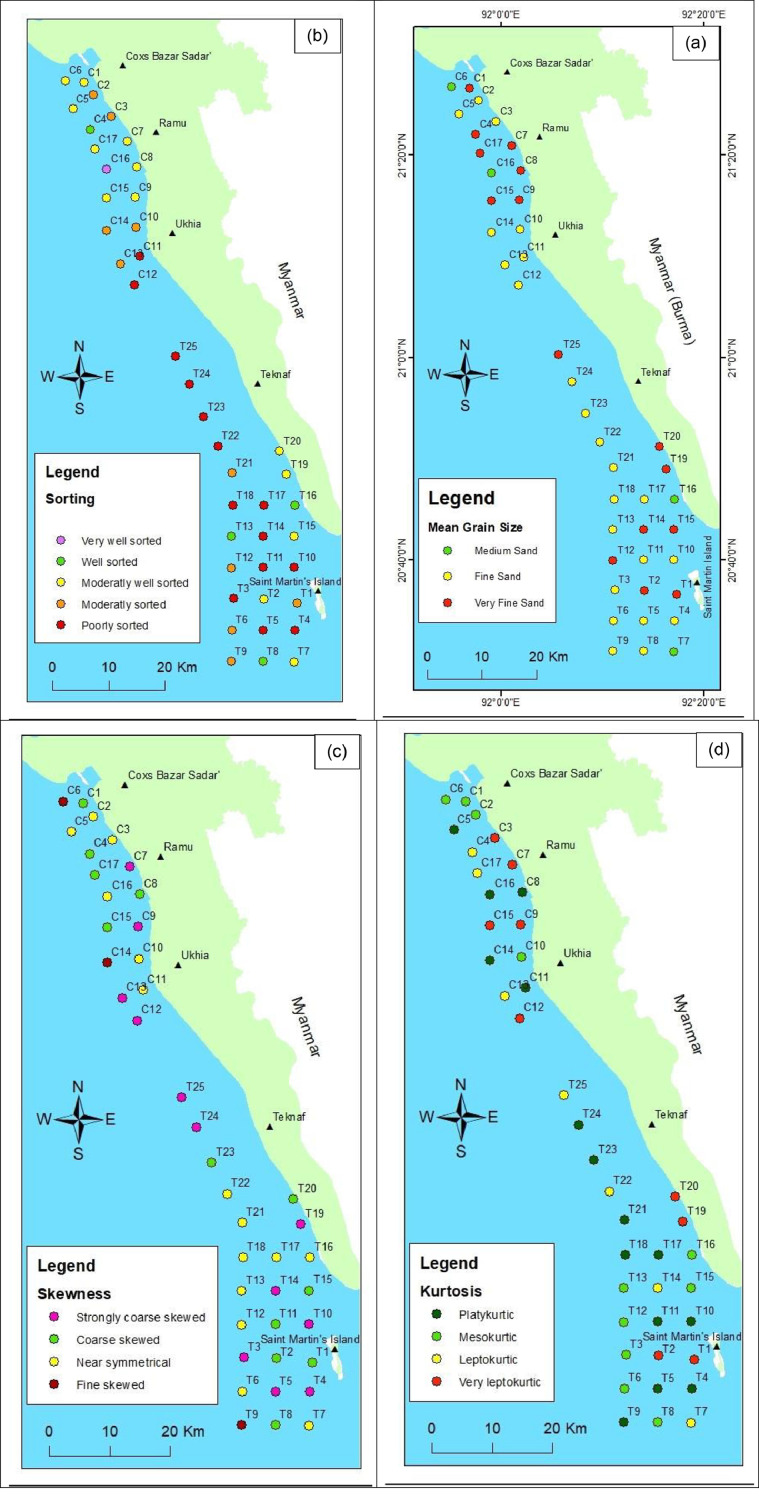
(a) Mean grain size**;** (b) sorting; (c) Skewness; (d) Kurtosis; classification of the samples.

Mean grain size is a descriptive parameter of grain size that measures the arithmetic average size of all the particles in a sample. It indicates the central tendency of a grain size distribution. The mean grain size of the samples mostly shows the fine sand size (2 to 3 mm). Of the 42 samples, 23 contain fine sand, 15 contain very fine sand, and 4 contain medium sand. The distributions of mean grain size are almost similar for all three coasts. The Teknaf coast sediment shows a dominant fine grain size. On the Teknaf and Saint Martin's Island coasts, samples show dominant finer sand than on the Cox's Bazar coast, where Cox's Bazar coast sediments are distributed with medium, fine, and very fine sand (Fig. 5a).

Grain-size sorting is a measure of the range of grain sizes present and the degree of spread or scatter of these sizes around the average or mean size. The inclusive graphic standard deviation is the mathematical expression for sorting. The sorting of the samples shows mostly poorly sorted sediment (14 samples), 10 samples are in moderately sorted sediment, 13 are in moderately well-sorted sediment, 4 are in well sorted, and 1 is in very well-sorted sediment. Most of the samples from the Teknaf coast and Saint Martin's Island are poorly sorted, whereas sediments from the Cox's Bazar coast are dominantly sorted. Also, the Cox's Bazar coast contains less poorly sorted sediments. The distribution of poor sorting of sediment is higher in the middle of the sampling area (C11, C12, T22 to T25) (Fig. 5b).

Skewness is a measure of symmetrical distribution, i.e., the proportion of coarse or fine fractions. A symmetrical curve with excess fine material shows a positive value, whereas one with excess coarse material shows a negative value; a zero value is indicated by a symmetrical curve. Positive skewness indicates a fine fraction abundant in the sediment [5]. The skewness shows dominants of near symmetrical value on all three coasts (*n* = 15). 12 samples show coarse skew, 12 are strongly coarse skewed, and 3 are fine skewed. The distribution of skewness values shows similarity along the three coasts (Fig. 5c).

The kurtosis expresses the packedness of the grain size distribution. The majority of the survey area's samples (*n* = 15) show platykurtic nature sediment, 11 show mesokurtic, 7 show leptokurtic, and 8 show very leptokurtic. Mesokurtic nature is dominant on the Saint Martin's Island coast. In terms of kurtosis, the majority of the samples show extreme values on the three coasts (platykurtic, very leptokurtic) (Fig. 5d). The extreme value of kurtosis can be used for the identification of the depositional environment [6].

## 3 Experimental Design, Materials, and Methods

Samples have been collected from the seafloor of the coastal and nearshore areas of the northern Bay of Bengal, located in the eastern coastal zone of Bangladesh. Samples were designed to be collected at 4–5 km intervals using fishing trawlers or boats according to a grid system. Samples are collected during the dry season (November–January) and pre-monsoon season (February–April) because sampling during the monsoon and post-monsoon is difficult using fishing trawlers or boats in rough sea conditions in the Bay of Bengal. A total of 42 samples were collected, and the sampling location was level. ‘T’ indicates the sample is near the Teknaf coast and Saint Martin's Island coast, and ‘C’ indicates the sample is near the Cox's Bazar coast. The sampling looks like there are some samples missing between samples T25 and C17 (in the middle of the map) (Fig. 1). It could be due to the hard or compact nature of the seafloor's sediment. Five times an attempt was made to capture samples from the gap area using the Van Veen grab sampler, but it failed. It is possible to assume that humans manually ran the sampler while sampling from the small floating fishing boat. Nonetheless, the other sample stations were successful using the same method. The 5 km grid interval sampling design was attempted to be maintained, but due to current and tidal influences, the fishing boat could not remain stable in the anchoring location while using a grab sampler.

25 samples were collected from the Teknaf coast and 17 samples from the Cox's Bazar coast. T1 to T15 (*n* = 15) samples were collected from February to March 2018, T16 to T25 (*n* = 10) samples from January to February 2019, and C1 to C17 (*n* = 17) samples from March to April 2019 (Table 1). The shallowest sample derived from the Cox's Bazar coast is 4 m in depth, and the deepest sample from the Teknaf coast is 23 m in depth. On the Teknaf coast, the shallowest depth (5 m) sample is T20, and the deepest sample (C13) of the Cox's Bazar coast is 18.30 m. The Ekman grab sampler has been attempted several times during sampling, but the sample was not retrieved with it.

**Table 1:** Sample location with acquisition date and coast name.

Sample ID	Latitude	Longitude	Acquisition date	Coast name
T1	20.60950	92.28833	25 February 2018	Saint Martin's coast
T2	20.61597	92.23503		
T3	20.61717	92.18612		
T4	20.56645	92.28448	26 February 2018	
T5	20.56607	92.23402		
T6	20.56675	92.18368		
T7	20.51580	92.28373	27 February 2018	
T8	20.51670	92.23333		
T9	20.51653	92.18333		
T10	20.66633	92.28350	01 March 2018	
T11	20.66633	92.23333		
T12	20.66583	92.18350		
T13	20.71617	92.18300	03 March 2018	
T14	20.71633	92.23417		
T15	20.71667	92.28343		
T16	20.76600	92.28500	09 January 2019	Teknaf coast
T17	20.76600	92.23500		
T18	20.76600	92.18500		
T19	20.81548	92.27088	10 January 2019	
T20	20.85290	92.25967		
T21	20.81776	92.18362	27 February 2019	
T22	20.86078	92.16136		
T23	20.90737	92.13850		
T24	20.96001	92.11538		
T25	21.00461	92.09395		
C1	21.44212	91.94748	13 March 2019	Cox's Bazar coast
C2	21.42243	91.96168		
C3	21.38762	91.99087		
C4	21.36754	91.95725		
C5	21.40052	91.92910		
C6	21.44560	91.91686		
C7	21.34850	92.01652	14 March 2019	
C8	21.30680	92.03181		
C9	21.25907	92.02858	21 March 2019	
C10	21.21086	92.02999		
C11	21.16440	92.03604		
C12	21.11913	92.02750		
C13	21.15244	92.00480		
C14	21.20548	91.98298		
C15	21.25741	91.98257		
C16	21.30385	91.98345		
C17	21.33548	91.96394		

The sediment has been placed in a geological bag and transported to the lab after sample collection. The sample was then subsampled to 100 g for dry sieve analysis after being dried in the open air for 30 days. Between sand and mud grain size 1 mm, 0.5 mm, 0.25 mm, 0.125 mm, 0.063 mm, and pan mesh sizes were used in a sieve machine (Advantech Dura Tape type) with 100 g dry samples that were shaken for 20 min. Using mesh size falls between sand and mud particle size. Then the measured sieved data is calculated for grain size analysis using Folk and Ward's [6] formula.

With the use of MS Excel analysis, the attribute table data values for the map were created using ArcGIS 10.5 [7] software

## Ethics Statements

This work did not involve human subjects, animal experiments or data collected from social media platforms.

## CRediT authorship contribution statement

**Md. Zakaria:** Conceptualization, Methodology, Software, Visualization, Investigation, Writing – original draft, Writing – review & editing. **Mst Tania Islam:** Project administration, Data curation, Software, Visualization, Validation, Writing – review & editing. **Sayeed Mahmood Belal Haider:** Project administration, Funding acquisition, Writing – review & editing.

## Declaration of Competing Interest

The authors declare that they have no known competing financial interests or personal relationships that could have appeared to influence the work reported in this paper.

## Data Availability

Coastal and nearshore sediment samples of the eastern coastal zone of Bangladesh (Original data) (Mendeley Data)
